# PDGF and PDGF receptors in glioma

**DOI:** 10.3109/03009734.2012.665097

**Published:** 2012-04-19

**Authors:** Inga Nazarenko, Sanna-Maria Hede, Xiaobing He, Anna Hedrén, James Thompson, Mikael S. Lindström, Monica Nistér

**Affiliations:** ^1^Department of Oncology-Pathology, Karolinska Institutet, CCK R8:04, Karolinska University Hospital Solna, SE-17176 Stockholm, Sweden; ^2^Karolinska Healthcare Research Biobank (KHRBB), Clinical Pathology/Cytology, Karolinska University Hospital, SE-17176 Stockholm, Sweden; ^3^(currently) Uppsala University, Rudbeck Laboratory, Department of Immunology, Genetics and Pathology, SE-751 85 Uppsala, Sweden

**Keywords:** Animal tumor model, brain tumor, cancer stem cell, glioma, neural stem cell, platelet-derived growth factor (PDGF), PDGF receptor

## Abstract

The family of platelet-derived growth factors (PDGFs) plays a number of critical roles in normal embryonic development, cellular differentiation, and response to tissue damage. Not surprisingly, as it is a multi-faceted regulatory system, numerous pathological conditions are associated with aberrant activity of the PDGFs and their receptors. As we and others have shown, human gliomas, especially glioblastoma, express all PDGF ligands and both the two cell surface receptors, PDGFR-α and -β. The cellular distribution of these proteins in tumors indicates that glial tumor cells are stimulated via PDGF/PDGFR-α autocrine and paracrine loops, while tumor vessels are stimulated via the PDGFR-β. Here we summarize the initial discoveries on the role of PDGF and PDGF receptors in gliomas and provide a brief overview of what is known in this field.

## Introduction

Gliomas are the most common form of primary malignancies of the central nervous system (CNS) mainly affecting adults. These tumors have a histological resemblance to different types of glial cells and are categorized into astrocytomas, oligodendrogliomas, oligoastrocytomas, and ependymomas, based on the predominant cell type(s) in the respective tumor (1).

Initial discovery and extensive studies of the epidermal growth factor (EGF) and transforming growth factor alpha (TGF-α), as well as the platelet-derived growth factor (PDGF) family, revealed their presence in glioblastoma cell cultures and tissues. These growth factors, by signaling via their cell surface tyrosine kinase receptors, EGFR and PDGFR-α and -β, were able not only to induce normal mitogenic and migratory responses, but when aberrantly activated they were also able to transform cells. Knowledge gained from *in vitro* experiments was transferred to *in vivo* systems, and several mouse models were created showing that gliomas can be induced by forced expression of PDGF-B in the brain. However, treatment with single tyrosine kinase inhibitors (TKIs) given to glioma patients has generally not been successful.

Ever since the initial discoveries, investigations on the molecular mechanisms and signaling pathways involved in glioma have provided a more detailed insight into the key events behind brain tumor initiation, maintenance, and progression and have resulted in significantly improved cancer drug discoveries. However, the prognosis for patients with aggressive brain tumors is still very poor. One intriguing hypothesis is that glioblastoma resistance to therapy and its rapid recurrence is due to the presence of a subpopulation of tumor cells displaying stem cell characteristics that are able to self-renew and propagate the whole tumor. In this context, we briefly discuss the concept of cancer stem cells (CSCs) in glioma.

## PDGF/PDGFR

Discovered as a serum growth factor for fibroblasts, smooth muscle cells, and glial cells, the PDGF family has become one of the most extensively studied growth factor families (2-6). PDGF-B and -A were discovered first, while PDGF-C and -D were identified decades later, based on gene homology searches. PDGFs are members of the evolutionary conserved family of structurally and functionally related PDGF/VEGF growth factors. The PDGF family now includes five dimeric proteins: PDGF-AA, -AB, -BB, -CC, and -DD. The mammalian PDGF genes are situated on different chromosomes and have mostly independent transcriptional regulation and thus distinct tissue- and time-specific gene expression patterns *in vivo*. However, the overlapping expression patterns of PDGF-A and -C suggest the possibility of common transcriptional regulatory mechanisms (6).

All PDGFs have a highly conserved growth factor domain, called the PDGF/VEGF homology domain. This domain is involved in forming inter- and intra-molecule disulphide bridges to form the PDGF dimers (7). In order to be activated, a short N-terminal extension present in PDGF-A and PDGF-B has to undergo intracellular proteolytic processing. PDGF-C and PDGF-D have a distinct protein domain as part of their N-terminal extension, called the CUB domain (7), preventing ligand–receptor binding until cleaved and are activated by extracellular proteases (8,9). The C-termini of PDGF-C and -D lack amino acid sequence extensions, while both PDGF-A and -B have a stretch of basic amino acids that are mainly involved in extracellular matrix (ECM) binding (10,11). There are two functionally distinct isoforms of the A-chain due to alternative splicing of exon 6, which encodes the C-terminal stretch: the short form of PDGF-A (PDGF-A_S_) lacks the positively charged retention motif and is freely diffusible, while the long form (PDGF-A_L_) can attach to the ECM with the help of its C-terminal tail (2,12,13). The exact role of PDGF-A_L_ and how it functionally differs from the shorter isoform is not well understood.

**Figure 1.**
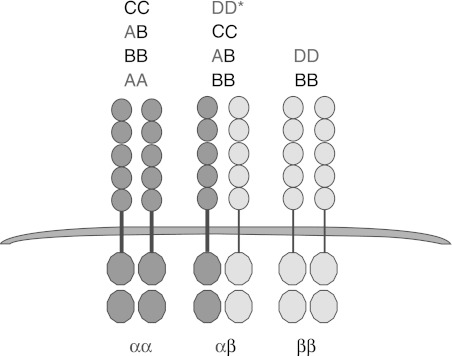
Receptor binding specificity of five dimeric PDGF ligands. *Ligand DD can activate αβ with lower specificity.

The five dimeric PDGF ligands act via the two cell surface tyrosine kinase (RTK) receptors, PDGFR-α and PDGFR-β (Figure 1). The PDGFRs have a common domain structure, including five extracellular immunoglobulin (Ig)-like domains and a split intracellular tyrosine kinase domain (6,14). As a result of ligands binding to the receptors, homo- and heterodimerization of the receptors occur. This in turn leads to transphosphorylation of the intracellular domains and receptor activation. Once activated, intracellular mediators dock to phosphotyrosine residues in the receptor, which leads to downstream activation of intracellular signaling pathways. The ability of each of the five dimeric PDGF ligands to bind and activate the two PDGF receptors is quite specific as summarized in Figure 1.

## Our early studies on PDGF in glioma

The initial discoveries on the role of PDGF and PDGF receptors in cancer came substantially from studies on human brain tumors. Our efforts in this field of research began in 1981 when Monica Nistér with Bengt Westermark was introduced to the vibrant and fruitful collaboration formed by Westermark, Wasteson, and Heldin. Carl-Henrik Heldin had purified and characterized human PDGF-AB from platelets (15). It had been shown that fibroblasts, smooth muscle cells, and glial cells were responsive to PDGF (3–5), and the outstanding question was whether PDGF was involved in human cancer.

Pontén and Westermark had established a large number of human glioma cell lines that were suitable to reveal if PDGF was produced by these cancer cells. One such biopsy was the U-343 MG biopsy for which a few clonal derivatives existed. Some cultures were GFAP^+^/astrocyte-like, while others had mesenchymal features (16,17). The strategy used by Nistér et al. was to screen conditioned media from U-343 MGa Cl2 cells using the ^125^I-PDGF-binding assay on fibroblasts to identify PDGF receptor competing activity. Much to our excitement, such activity was detected, and a following protein purification confirmed the presence of a secreted PDGF-like activity (18). Further purification and characterization attributed this activity to a 31-kDa dimeric protein with structural, functional, and immunological similarities to PDGF (19).

At this time, Wong-Staal and co-workers had already reported a human gene homologous to v-*sis*, simian sarcoma virus (SSV) oncogene (20). But it was not until PDGF was sequenced in 1983 that the homology between the PDGF B-chain and p28v-*sis* was revealed and PDGFB (c-*sis*) was identified as a proto-oncogene (21). The cell surface receptor for PDGF, now known as the PDGF β-receptor, was found to be a tyrosine kinase (22). Similarly, EGFR was a tyrosine kinase, homologous to the v-*erbB* oncogene (23). These groundbreaking findings brought a lot of interest to the field, which was spurred by the homology between p28v-*sis* and PDGF-B and by the fact that SSV originally had been isolated from a fibrosarcoma in a woolly monkey (24).

When tested on fibroblasts and other normal cell types, the PDGF protein isolated from glioma-conditioned media showed receptor-binding characteristics and biological effects different from those of PDGF-BB and -AB. It inhibited PDGF-BB-induced chemotaxis and actin reorganization and had a lower mitogenic activity than PDGF-AB purified from platelets (25). These findings were initially challenged but could later be confirmed by other groups and explained by the presence of a second receptor for PDGF. Further work identified the purified glioma-derived activity as PDGF-AA. Thereafter it was discovered that glioma cells produced PDGF-AA, -AB, and -BB dimeric proteins (26).

Human PDGFA was cloned by Betsholtz et al. after generating a cDNA library from a clonal glioma cell line, U-343 MGa Cl2:6, that produced high amounts of PDGF receptor-competing activity. The isolated cDNA included two splice variants of PDGF-A, a short form (PDGF-A_S_) and a long form (PDGF-A_L_), which in the latter case includes a 69 base pair (bp) extension from exon 6, that codes for a C-terminal stretch of basic amino acids (13,27,28). While the PDGFB gene was localized on chromosome 22, the PDGFA gene was found to be residing on chromosome 7.

By sub-cloning cells from the U-343 MGa cell line, Nistér et al. identified an extensive clonal variation in the amount of PDGF secreted by glioma cells and also a clonal variation in the capacity of glioma cells to bind ^125^I-PDGF (29). The cells growth rate in serum-free medium correlated to the amount of PDGF produced by them. The most prominent PDGF producers were also ‘immature', tightly growing cells, while clones with large, glia-like cells showed the highest ^125^I-PDGF-binding capacity. Thus, there was a negative correlation between the levels of PDGF secreted from the cells and their capacity to bind the factor. By Northern blot analysis, using a PDGFR-β cDNA sequence as a probe, Nistér et al. identified PDGFR-β mRNA in some of the glioma cell lines and clones. Interestingly, the U-343 cell clones with the highest capacity to bind ^125^I-PDGF-AA displayed a different-sized PDGF receptor mRNA. Claesson-Welsh et al. were then able to identify the second PDGF receptor, PDGFR-α, expressed in U-343 MGa 31L cells (30). Further interest came when the PDGFR-α cDNA was cloned (31,32). Using ^125^I-binding assays, the presence of both PDGF receptors and EGF receptors on human glioma cells was also described (33).

With the new cDNA, mRNA, and antibody reagents available at Heldin's laboratory that recognized PDGFs and their receptors, investigation into distribution was commenced using human brain tissue, in collaboration with Keiko Funa. Using mRNA *in situ* hybridization, high levels of both PDGF-B and PDGFR-β mRNA were found on the characteristic glioblastoma vasculature, while the glial tumor cells expressed more PDGF-A mRNA(34). This was confirmed by a study of all glioma grades, where it was also observed that the PDGFR-α was present on glial tumor cells but not on the vasculature. Much to our excitement, it was shown that PDGFRA was overexpressed already in low-grade diffuse astrocytomas and further enhanced in high-grade tumors. The findings indicated the presence of PDGF autocrine/paracrine loops stimulating glioma cells via the α-receptor, and a PDGF-B autocrine loop stimulating the vasculature via the β-receptor (35). PDGFRA expression was examined in a larger number of glioma samples of different malignancy grades (36). Increased levels of PDGFR-α mRNA were observed in all grades, but glioblastoma expressed the highest levels. Only 16% of glioblastomas showed PDGFRA amplification, which suggested that other mechanisms were responsible for PDGFRA overexpression in the majority of tumors. The latter has also been indicated by more recent studies (37,38). PDGFRA expression correlated to loss of heterozygosity (LOH) on chromosome 17p, where the TP53 gene is localized (36). PDGFRA amplification had been described by Fleming et al. (39) and was confirmed by Hermanson et al. (36). More recently it has been reported that PDGF-C and -D are also present in human glioma (40).

The testing of the first generation of TKIs, that were more or less specific for the PDGF receptors, was disappointing. The inhibitors did not significantly alter the *in vitro* growth of glioma cells. Moreover, whereas retrovirally introduced PDGF-B was able to transform fibroblasts *in vitro*, PDGF-A could not (41). Whether this could be attributed to the presence or absence of the C-terminal stretch of basic amino acids was unclear at the time. In addition, the inhibitory effect of PDGF-A on PDGF-B-mediated cell proliferation and motility was puzzling, and the significance of PDGF-A/α-receptor signaling in tumor development was in question.

Although autocrine stimulation was extensively studied at the time, its significance in cancer was difficult to finally prove. However, the transforming effect of PDGF in glioma cells was indicated by the inhibitory effects of PDGF-BB antibodies (42) and by studies using a truncated PDGF β-receptor (43). Even now, clinical trials using TKIs fail to show significant therapeutic effects in patients (44,45).

Our recent investigations, performed in collaboration with Arne Östman, Johan Holmberg, and Jonas Muhr, have shed some light on the glioma cell resistance to TKI inhibitors. Using tumor material explanted from a number of human glioblastomas, two different subtypes of glioma cultures, type A and type B, were identified. Whereas type B cultures showed mesenchymal features and were responsive to mono-treatment with the PDGFR inhibitor imatinib, and also to IGF-1R inhibitor, NVP-AEW541, the type A cultures displayed stem cell-like features, including *in vitro* self-renewal and neurosphere formation, and *in vivo* tumor growth. These type A cultures were non-responsive to mono-treatments but were growth-inhibited when exposed to a combination of the two inhibitors. Furthermore, the cells' resistance to mono-treatment was dependent on the presence of transcription factor SOX2 (46). In a study by Holmberg and Muhr, SOX2, OCT4, NANOG, and KLF4 transcription factors were visualized in high-grade human glioma tissues using immunofluorescence techniques (47). These proteins were studied because they are able to induce pluripotent stem cells (iPS cells) from somatic cells (48), and their presence in glioma indicates a stemness phenotype. The stem cell features may be part of glioma cells' resistance to TKIs, and a combined treatment strategy targeting key stem cell regulatory mechanisms and growth factor signaling mechanisms promises to be a useful therapeutic approach.

## The role of PDGF during development

The functional significance of the described diversity in PDGF-A/B ligands and receptors, encoded by the different genes, became evident from the phenotypes of a number of genetically modified mice generated by the groups of Christer Betsholtz and Phil Soriano (for a review see (49)). In addition, the transcriptional regulatory mechanisms also differ, thus providing a basis for cell-, tissue-, and time-specific activities *in vivo*. This diversity turned out to be even higher when PDGF-C and -D were identified (8,9).

A large number of gain- and loss-of-function mutations in Pdgf and Pdgfr genes have been created in mice (50-54). The developmental defects found in these mice underscore the importance of PDGF ligands and receptors in normal embryonic development. PDGF-B and β-receptors are essential for normal blood vessel development, while PDGF α-receptors are required for neural crest development. PDGF-B stimulates pericytes (55) and is involved in formation of glomeruli of the kidney. PDGF-A is required for the normal development of lung alveoli (50,56), intestinal villi, mesenchymal dermis, and hair follicles. Studies in genetically modified mice, where the cytoplasmic signaling domains of the two PDGF receptors had been swapped, demonstrated that PDGF receptors are partly interchangeable during development and mediate very similar cellular responses (57). However, PDGFR-β seems to have a more important intracellular signaling capacity in the vasculature, since mice with an introduced PDGFR-α signaling domain exhibited vascular defects (58).

PDGFs are also important in adulthood, as they are involved in wound healing, including wound healing in the CNS (59). However, excessive or aberrant expression of PDGFs can lead to pathological responses such as atherosclerosis, fibrosis, and tumorigenesis (6).

## The role of PDGF in the central nervous system

Some insights into the role of PDGFs in the CNS were obtained before genetic mouse models were available. Initially, our knowledge on the role of PDGF-B and PDGFR-β in the CNS was mainly based on their expression patterns in the brain. PDGF-B is present in embryonic as well as in adult neurons (60). PDGFR-β was detected in neurons, and PDGF was shown to mediate neuroprotective functions after injury (61-63). PDGFR-β is also present on pericytes (64,65).

A series of *in vitro* studies determined that PDGF-A is produced by neurons and astrocytes (66-68) and acts as a mitogen for oligodendrocyte progenitor cells (OPCs) (67,69,70). Oligodendrocytes differentiate postnatally from PDGFR-α--positive OPCs. Their continued proliferation and migration in the CNS depends on PDGF-A signaling through PDGFR-α (71,72). In the absence of PDGF-A, postnatally surviving mice develop tremor due to severe hypomyelination (71,72). Finally, the amount of PDGF-A available controls the number of OPCs not only during embryogenesis, but also in the adult brain (73,74).

Recent *in vitro* experiments demonstrated the ability of PDGF-A to induce embryonic Nestin^+^ neural progenitor cells towards becoming NG2^+^ oligodendrocyte precursors (75). In addition, direct stimulation with PDGF-A of cells in the adult lateral ventricular wall subventricular zone (SVZ), induces PDGFR-α--positive neural stem cells (NSCs) to give rise to oligodendrocyte lineage cells, but not neuronal lineage cells (76,77).

## Human glioma tumors

Gliomas are the most common primary tumors of the CNS mainly affecting adults. They are categorized into astrocytomas, oligodendrogliomas, oligoastrocytomas, and ependymomas, reflecting their histologic appearance. In a clinical setting, the malignancy grade of the tumor is an essential factor to help predict the outcome of the patient and the choice of therapy. According to the World Health Organization (WHO) grading scale for tumors of the nervous system, lesions with no atypia/low proliferative activities are denoted as grade I, quite often curable upon surgical removal. Once the lesion is infiltrative it is designated as grade II, and tumors of this grade can progress to higher grades. Grade III are lesions with accelerated mitotic activity and nuclear atypia/anaplasia. Grade IV is the most malignant tumor grade with a fatal outcome. Grade IV tumors often present with infiltration into surrounding tissue, high mitotic activity, characteristic necrotic areas, and extensive microvascular proliferations (78).

Diffuse astrocytomas grade II and III may progress to grade IV, which is then referred to as secondary glioblastoma (GB). However, the majority of GB tumors develop *de novo*, with no previous history, and are referred to as primary glioblastomas (78). The primary and secondary GBs exhibit the same histopathologic characteristics, even though they differ in both genetic changes and clinical history (79).

## Activated growth factor pathways in glioma

A frequent hallmark of malignant gliomas is activation of RTK signaling pathways, most commonly caused by EGFR mutation/amplification or PDGFR amplification/overexpression. EGF/TGF-α and PDGF proteins exert their activity by binding to and activating cell surface tyrosine kinase receptors, which leads to receptor dimerization, transphosphorylation, and subsequent activation of intracellular signaling pathways, such as PI3K/AKT and RAS/MAPK.

Amplification of EGFR is found in about 43% of primary GBs and is associated with EGFR overexpression. However, EGFR overexpression has rarely been found in secondary GBs (79-83). Furthermore, 70%–90% of all GBs with EGFR overexpression have rearrangements of the gene (82). The most widespread mutated variant of EGFR is EGFRvIII, which contains a 267-bp deletion of exons 2–7 in the extracellular domain, resulting in ligand-independent activation of the receptor (79,83-85).

Overexpression/hyperactivity of PDGF ligands and receptors are frequent events in human gliomas of all grades (33,35,86,87), and their expression pattern in tumors suggests the presence of autocrine and paracrine stimulatory loops (36,40). Amplification of PDGF and PDGFR genes is not as common as the amplification of EGFR (36,39) and occurs only in 11% of GBs. However, this still makes PDGFRA the second most frequently amplified RTK gene in these tumors (82). Activating rearrangements of PDGFRA in GBs is very rare. Previously, only two reports described an in-frame deletion of the Ig-like domain, the PDGFRA^Δ8,9^ mutant and a mutation in the C-terminal of PDGFRA (88,89). Recent sequencing analysis of GBs has identified several point mutations in the Ig-like domains (90), and another study discovered a gene fusion between PDGFRA and the kinase insert domain receptor (KDR/VEGFR2) gene (91). Ozawa and colleagues have also demonstrated that the previously discovered PDGFRA^Δ8,9^ mutant is present in 40% of GBs with PDGFRA amplification.

Alterations in other RTK genes have been reported, including ERBB2/HER2 mutations and MET amplifications in 8 and 4% of GBs analyzed, respectively (82).

High levels of active RAS have been reported in high-grade astrocytomas, but unlike in many cancers mutated RAS is rarely present in malignant gliomas (2%) (82). Neurofibromin-1 (NF1) is a tumor suppressor and a negative regulator of RAS. Mutations of *NF1* have been linked to the hereditary condition neurofibromatosis type-1, where patients are predisposed to glioma development (92). NF1 was recently found to be mutated in 18% of glioblastomas (82).

Activation of PI3K/AKT signaling can be achieved by loss of the tumor suppressor gene PTEN or by mutations in PIK3CA. PTEN is a direct antagonist of the activity of PI3K. *PTEN* loss is rare in low-grade gliomas, but mutations and deletions are found in 50% of high-grade gliomas (93) and are associated with poor patient survival. As a result, inactive PTEN leads to AKT hyperactivation, which in turn triggers downstream pathways supporting cellular growth (through mTOR) and proliferation (through inhibition of GSK3-β) (94).

## Loss of cell cycle regulation in glioma

The tumor suppressor protein p53 is a major regulator of multiple cellular responses, including those induced by DNA damage, oncogene activation, and hypoxia (95). In GBs, TP53 is found to be mutated in 35% of all cases (82). It has been described that somatic TP53 mutations are more common in low-grade astrocytomas and secondary GBs than in primary GBs and that the spectrum of mutations is different (79,96). However, recent studies have confirmed that TP53 mutations are also prevalent in primary GBs (97,98). In addition, chromosome 17p is an early and frequent target for LOH in both low-grade and high-grade gliomas (99,100). Inactivation of p53 can also occur through other mechanisms such as viral infection, loss of ARF, or overexpression of MDM2 (101). The progression from G1 to S phase in the cell cycle is controlled by the p16INK4A/CDK4/RB1 pathway. Genetic alterations involving this pathway are found in 78% of glioblastomas (82).

## Molecular-genetic subclassification of high-grade glioma

Previous clinical and genetic studies of GBs described two subtypes, primary and secondary. However, recent genomic studies have provided more detailed information about the molecular mechanisms involved in glioma initiation and progression and have identified new subgroups based on glioma molecular signatures (82,97,102-104) that are described as classical, mesenchymal, proneural, and neural subtypes. Among these subtypes, the proneural subtype is characterized by PDGFRA amplification and loss or mutation of TP53, CDKN2A, and PTEN. Mutation of the isocitrate dehydrogenase 1 gene (IDH-1) (see below) and activation of PI3K and PDGFR-α pathways are also frequent characteristics of this subtype. GBs of the secondary subtype have gene expression profiles corresponding to those of neuronal (SOX2, DCX, etc.) and oligodendrocytic (PDGFRA, OLIG2, etc.) progenitor cells. These findings have been comprehensively discussed (90,105,106). In addition to distinct genomic profiles, the classical, mesenchymal, and proneural subtypes vary in their biological behavior and response to adjuvant treatments (107).

Recent results from GB tumor DNA sequencing studies also revealed previously unknown important genetic changes. Spontaneous mutations of *IDH-1* and *IDH-2* appeared as strong prognostic indicators in anaplastic astrocytoma and secondary GB. In patients with secondary GB, mutation of *IDH-1* is linked to a median survival of 31 months compared to 15 months for the group of patients with wild-type (WT) *IDH-1* (104). Importantly, these mutations also occur together with *TP53* mutations in lower-grade tumors.

## Neural stem cells

A common belief of classical neuroscience was that once development was completed, no new neurons were produced. However, this view has changed, and in the 1960s the first evidence of adult neurogenesis appeared from studies in rat brain (108). During brain development, neuroepithelial stem cells situated in the ventricular zone of the lateral ventricle wall give rise to neurons and glia. Even though it was thought that neurogenesis is mostly completed by birth, it continues throughout life. Cells, commonly known as neural stem cells (NSCs), have a capacity to self-renew and differentiate along multiple lineages, contributing to tissue maintenance and regeneration in case of injury in the adult CNS (109,110).

There are two brain areas in mice where adult NSCs are known to reside, the SVZ of the lateral ventricle and the subgranular zone (SGZ) of the dentate gyrus in the hippocampus. The largest source of NSCs in the adult mammalian brain is the SVZ, which is described as a thin layer of proliferative cells in the lateral wall of the lateral ventricle (109,111-113). Adult NSCs are not unstructured undifferentiated cells; they show features of differentiated astrocytes and express glial fibrillary acidic protein (Gfap). This subpopulation of Gfap^+^ cells in the SVZ (B cells) produces a transit-amplifying cell population (C cells) that then gives rise to migratory neuroblasts (A cells) and OPCs. It has been shown that adult NSCs are derived from radial glia, the stem cells of the developing brain, which in turn are derived from the neuroepithelium (114). In addition to Gfap, NSCs are characterized by expression of Nestin (112). Sox2 is also found in NSCs of the SVZ (115) and SGZ (116) and is required to maintain the immature state of NSCs and to preserve their capacity to proliferate and generate neurons (117,118).

There are several architectural elements contributing to adult neurogenesis, such as localization of NSCs near the cerebrospinal fluid, close association with blood vessels, a rich extracellular matrix (ECM), specialized basal lamina, and widespread cell-to-cell interactions (119,120). The endothelial cells of blood vessels release factors that promote the self-renewal of NSCs (121,122).

## Regulatory pathways in neural stem cells and their relation to brain tumors

It was demonstrated that a fraction of SVZ stem cells express PDGFR-α (76) and that EGFR is expressed in type B and type C cells (123). It has also been shown that NSCs proliferate in response to both PDGF and EGF *in vitro* (110,112,124,125), and EGF is used in cultures to keep NSCs in an undifferentiated state (110). Infusion of EGF into the brain results in a significant amplification of endogenous SVZ precursor cells (123,124,126) and promotes oligodendrogenesis (127). When active EGF receptor was introduced into *Ink4a/Arf*-deficient mice, it caused dedifferentiation of astrocytes, and high-grade gliomas developed from both dedifferentiated astrocytes and NSCs carrying the same genetic alteration (128). PDGF also has a dedifferentiating effect on mouse astrocytes (129), and infusion of PDGF-A into the lateral ventricle of adult mouse brain can induce the proliferation of PDGFR-α--positive NSCs that may form glioma-like lesions (76).

The tumor suppressor p53 plays an important role during development of the CNS by inducing apoptosis of neurons and neural progenitors to adjust the cell number (130), and a subset of *Trp53*-deficient mice develop exencephaly (131,132). P53 is present in the adult mouse lateral ventricle wall SVZ and regulates NSCs proliferation, apoptosis, and self-renewal. Gene expression profiling identified several genes that were down-regulated in *Trp53 null* NSCs *in vitro*, such as the cell cycle regulatory factors p21 and p27 (133).

## Cancer stem cells; heterogeneity of glioma cells

The heterogeneity of glioblastoma cells has become increasingly appreciated as a significant factor, allowing for paracrine stimulatory mechanisms in tumors (134). It has also been shown that within some tumors there is a subset of cancer cells (CSCs) with stem cell-like features: unlimited self-renewal, multipotency, and tumor-initiating capacity *in vivo*. These cells may give rise to a number of new cell types in the tumor and maintain the neoplastic growth over time. CSCs have been described in brain tumors (135-140).

**Figure 2.**
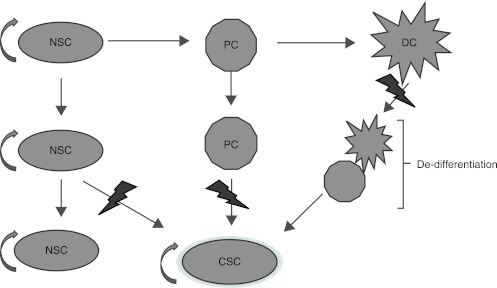
Three possible ways for a cancer stem cell (CSC) to arise: a neural stem cell (NCS) acquires a mutation; a progenitor cell (PC) acquires two or more mutations; or a fully differentiated cell (DC) undergoes several mutations that transform and drive it back to a stem-like state.

A high rate of recurrence and resistance to treatment are major characteristics of malignant gliomas. It has been reported that brain CSCs have the capacity to survive treatment and give rise to a new tumor with characteristics of the original tumor (141). Since the adult brain has only a limited population of proliferating cells that are able to accumulate the several mutations required for transformation into a cancer cell, the adult NSCs/progenitor cells have been suggested as candidate sources for brain CSCs (Figure 2). In addition, it has been suggested that tumorigenic events can lead to dedifferentiation of a mature cell and thereby gain of stem cell properties (Figure 2) (128,129,142). NSCs are relatively quiescent cells, whereas glioblastoma cells are highly proliferative. It is therefore possible that the majority of tumor cells represent rapidly amplifying progenitors rather than stem cells.

Similar to NSCs, brain CSCs are located in a special microenvironment. They are found in close proximity to blood vessels, often in hypoxic areas (143). They have been shown to stimulate angiogenesis by secreting VEGF (144), and in return they receive a constant supply of nutrients and oxygen (145). Glioma cells migrate along the tumor vessels into the surrounding tissue. Other components of the perivascular niche, such as adhesion to the surrounding cells or ECM are important for CSCs maintenance. L1CAM, a cell surface molecule that intervenes in cell–ECM and cell-to-cell interactions, was found to be highly expressed in glioma CSCs (146,147).

## PDGF-driven glioma models

To address the functional role of PDGF in brain tumors, several experimental models have been created to induce gliomas, mostly in mice by forced expression of PDGF. In general, overexpression of PDGF in the brain leads to excessive production of OPCs and, if in a permissive setting, results in mainly oligodendroglioma-like tumors.

The first attempt to model ‘PDGF-induced' glioma was made by Deinhardt (148), who injected SSV, with the v-*sis* oncogene, into the brains of newborn marmosets. This resulted in gliomas, morphologically indistinguishable from human tumors, described in a review by Nistér and Westermark (149). Many years later, when a PDGF-B-encoding retrovirus was used for injection in newborn mice, highly malignant brain tumors developed in 40% of all animals, and further *in vitro* experiments showed that autocrine PDGF stimulation was an important step in their generation (150,151).

As an alternative approach, the RCAS/TV-A model system was developed for brain tumor studies (152,153) and has been successfully used to assess the significance of PDGF and downstream signaling mechanisms in glioma. Tumor progression can be enhanced in the PDGF-induced glioma models by combination of another genetic aberration such as loss of *Ink4a/Arf*, *Trp53*, or *Pten* (154-156). However, a disruption of the homology-directed DNA repair mechanism, by introduction of RAD51, decreases PDGF-B-induced tumor incidence and progression (157).

In order to test the tumorigenic potential of adult glial progenitors, PDGF-B was retrovirally expressed in rat corpus callosum, leading to transformation of NG2-positive OPCs and development of GBs (158). Injection of retroviral PDGF-B in newborn rats causes a shift in the differentiation fate of the NSCs, generating more pdgfr-α/NG2/Olig2-expressing OPCs that do not differentiate into mature oligodendrocytes (159). Moreover, excessive expression of PDGF-B in neural progenitor cells forced their respecification towards the oligodendroglial lineage with development of highly malignant oligodendroglial tumors in mice (160,161).

## Different capacities of PDGF-B and PDGF-A_L_ in brain tumor development

To investigate the roles of PDGF-B and PDGF-A in brain tumor development, two transgenic mouse models were generated in which overexpression of human PDGF-B (162) and PDGF-A_L_ (163), respectively, was induced in astrocytic cells of the brain by a human GFAP promoter fragment. Both these PDGF proteins have the C-terminal motif, attaching them to the ECM, but while PDGF-B binds to both α- and β-receptors, PDGF-A_L_ binds only to the α-receptor. The GFAP promoter fragment used is active during embryogenesis, in radial glial cells of the telencephalon and in the cerebellar anlage, and stays active postnatally in astrocytic cells, including activity in astrocytic cells of the adult SVZ. The effects of PDGF-A_L_ and PDGF-B were found to be different.

The PDGF-B-overexpressing transgenic mice did not develop brain tumors, and the mice were phenotypically normal. They were therefore crossed with *Trp53 null* mice to obtain PDGF-B/p53-null offspring. In this way, two aberrations commonly present together in human gliomas could be combined. On the *Tp53 null* background, two PDGF-B transgenic mouse lines developed brain tumors at high rates, 68 and 43%, and tumors occurred between 2 and 6 months after birth. They spread diffusely throughout the brain tissue and displayed microvascular proliferations as well as necroses lined by palisading cells. Thus, morphologically the tumors had the characteristics of human GB. According to previous findings from human brain tumors, PDGFR-α is localized on glial tumor cells, and PDGFR-β on the vasculature (35). This was also the case in the PDGF-B/p53-null tumors that were generated. The majority of glioma cells proper, but not the tumor vasculature, expressed the transgene, and together this indicated the possibility of both autocrine and paracrine stimulatory loops similar to those in human tumors (162). Small Gfap^+^/Nestin^+^ SVZ lesions were present in the lateral ventricular wall and were suggested to represent a point of origin. Due to the variable location of the tumors and the fact that the PDGF-B transgene was produced by astrocytic cells throughout the brain, such as in the glia limitans, and not only in the SVZ, another suggested possibility was that astrocytes dedifferentiated and transformed into tumor cells. Larger lesions tended to lose Gfap but gain Nestin positivity and developed the characteristic glioma vasculature and necroses. Thus, sustained overexpression of PDGF-B in astrocytic cells can, on a *Tp53 null* background, lead to uncontrolled expansion of Pdgfr-α^+^ glial progenitor cells and initiate the development of aggressive GB-like brain tumors.

Whereas the PDGF-B overexpressing transgenic mice were phenotypically normal, with survival times that did not differ from WT mice, PDGF-A_L_-overexpressing mice developed a characteristic, lethal phenotype, after approximately 6 weeks (163). A microscopic analysis of brain tissue showed an increased cellularity due to a highly proliferating, expanding mixed population of astrocytic and OPC-like cells, that spread diffusely throughout the brain and that was also concentrated in distinct brain areas, which overlapped with the areas of transgene activity, including: the roof of the lateral ventricles, in corpus callosum, on the outer surface of the brain, and in the cerebellum. Closely associated with the Gfap^+^ astrocytic cells were these OPC-like cells with the presence of the oligodendrocyte markers, Pdgfr-α/Olig2/NG2.

A few of the PDGF-A_L_ mice presented with very advanced lesions, in the form of a heavy diffuse infiltration of cells throughout the whole brain, comprising mostly of white matter areas and also in the form of pial outgrowths with excessive masses of mitotic and atypical cells. Also here, the cell population was mixed, with Gfap^+^ and Pdgfr-α^+^/Olig2^+^/NG2^+^ cells. These lesions were associated with long, angulated and thick-walled capillary structures, strongly positive for Pdgfr-β. Thus, these lesions were found to be similar to human anaplastic oligoastrocytomas (grade III).

The majority of Olig2^+^/Pdgfr-α^+^ cells in the examined PDGF-A_L_ brain sections did not express Gfap or the transgene. This observation could be due to the fact that the PDGF-A_L_ transgene produced by NSCs/astrocytic cells stimulated OPCs in a paracrine fashion, or indirectly via changing the cell fate of the NSC/astrocytic progenitor. Additional experiments are required to resolve this issue.

Because PDGF-A_L_ has a C-terminal retention motif that makes it associate with ECM and possibly with the surface of the producing cell, one would expect that its area of action is rather limited. Pdgfr-α is normally expressed by glial progenitor cells (164,165), and as discussed previously PDGF-A is a potent mitogen for OPCs (71,72,74). PDGF-A can also influence cell fate decisions of cells located in the adult SVZ by inducing them to become OPCs rather than neurons. These cells are sensitive to alterations in the PDGF pathway and can form hyperplasias in response to PDGF-A_S_, as mentioned previously (76).

In summary, our research work and that of others demonstrate that overexpression of PDGF-A_L_ in astrocytic cells can induce oligoastrocytoma-like lesions at a low rate. Hyperactive production of PDGF-A_L_ in the brain has distinct and strong effects that are different from previously described effects of PDGF-A_S_ and PDGF-B. PDGF-B, in spite of it being expressed in the SVZ by astrocytic cells and carrying the C-terminal retention motif similar to PDGF-A_L_, resulted in a normal phenotype. It cannot be excluded that the difference was a matter of local concentrations of growth factor. However, the fact that concurrent loss of p53, a known regulator of stem cell self-renewal resulted in heavy amplification of a more undifferentiated Nestin^+^/mostly Gfap^-^ neural cell type, forming GB-like tumors, suggested a difference in the capacity of progenitors to respond to the PDGF proteins.

In conclusion, the role of PDGF-A that was shown in this model system is consistent with the PDGFRA overexpression observed in human low-grade gliomas, whereas the role of PDGF-B was to promote the full-blown vascular proliferations that are characteristic of human GBs. The loss of p53 function may in addition have allowed for enhanced self-renewal of early neural progenitors. SVZ cells are known to be affected by loss of p53 (166).

We speculate that in the human situation, the early and almost ubiquitous TP53 mutations/deletions on chromosome 17p observed in low-grade astrocytic glioma may similarly allow for the emergence of stem cell features during glioma progression. Additional experiments are required to resolve this issue.

## Final comment

It has been an honor to participate in the research work on platelet-derived growth factors. It is also stimulating to think of the extensive technical developments and the progress made in the fields of normal brain development and glioma biology over the last 30 years, and look forward to further developments that may improve the therapy of brain tumor patients.
